# Dr. Emily H. Wells

**Published:** 1913-10

**Authors:** 


Dr. Emily H. Wells, Woman's Medical College of the New York Infirmary for Women and Children, 1873, died at her home in Binghamton, August 20, aged 72. She was at one time President of the Broome Co. Medical Association.

